# Serum Leptin Is Low and Linked to Obesity and Insulin Resistance in Bangladeshi Youth-Onset Type 2 Diabetes Patients: A Cross-Sectional Study

**DOI:** 10.7759/cureus.85562

**Published:** 2025-06-08

**Authors:** Indira Roy, Nusrat Sultana, Mashfiqul Hasan, Tahseen Mahmood, Md. Shayedat-Ullah, Muhammad Abul Hasanat

**Affiliations:** 1 Endocrinology, Bangladesh Medical University, Dhaka, BGD

**Keywords:** bangladesh, insulin resistance, leptin, obesity, t2dm, type 2 diabetes mellitus, youth-onset t2dm

## Abstract

Background

Youth-onset type 2 diabetes mellitus (T2DM) is an escalating global public health concern, mostly driven by increasing obesity and insulin resistance. Leptin, a key adipokine regulating appetite, energy balance, and metabolism, has been implicated in the pathogenesis of diabetes. However, its role in youth-onset T2DM remains unclear, with conflicting findings.

Objective

This study aimed to measure and compare circulating leptin levels between Bangladeshi young individuals with a clinical diagnosis of new-onset T2DM and normal glucose tolerance (NGT), and to investigate the associations of leptin with obesity markers, glycemic parameters, and insulin resistance.

Methods

This cross-sectional study involved 36 youth (aged 13-30 years) with a clinical diagnosis of new-onset T2DM and 36 age-matched individuals with NGT attending the young diabetes clinic of Bangladesh Medical University (BMU), Bangladesh, during 2017-2018. Anthropometric measurements were recorded, and fasting blood samples were analyzed for glucose, insulin, and leptin levels by glucose-oxidase, chemiluminescent immunoassay, and enzyme-linked immunosorbent assay, respectively. Insulin resistance was assessed by the homeostasis model assessment-insulin resistance (HOMA-IR). Body mass index (BMI) was calculated and both the T2DM and NGT groups were dichotomized to non-obese (including normal weight and underweight; for adults, BMI <23 kg/m^2^, for children <85^th^ percentile) and obese (including overweight and obesity; for adults, BMI ≥23 kg/m^2^, for children ≥85^th^ percentile) for subgroup analysis.

Results

Leptin levels were significantly lower in the T2DM group compared to the NGT group (median (IQR): 1.6 (0.2-6.6) vs. 11.0 (3.5-18.4) ng/mL; p < 0.001). Within subgroups, non-obese individuals with T2DM had lower leptin levels than both non-obese and obese NGT individuals, while obese individuals with T2DM had lower leptin levels than obese NGT individuals. Leptin levels positively correlated with BMI (ρ = 0.45, p < 0.001) and waist circumference (ρ = 0.28, p = 0.019) but negatively with glycemic parameters and HOMA-IR. In multivariate analysis, higher BMI and normoglycemic status were independent predictors of elevated leptin levels.

Conclusions

Despite a positive correlation with obesity markers, leptin levels were significantly lower in newly diagnosed youth-onset T2DM compared to NGT and had a negative correlation with insulin resistance.

## Introduction

In recent years, youth-onset type 2 diabetes mellitus (T2DM) has emerged as a global public health concern, with its prevalence steadily increasing [1,2]. South Asians are currently facing an epidemic of diabetes in young individuals, likely driven by a sedentary lifestyle, unhealthy dietary habits, and rising obesity rates, all of which contribute to insulin resistance [3]. The diabetogenic process begins in fetal life, where low birth weight and poor nutrition interact with postnatal environmental factors, including physical inactivity and an energy-dense diet, to produce an insulin-resistant phenotype in children and adolescents, ultimately leading to T2DM [4]. Overweight and obese children are at a higher risk of remaining obese into adulthood and are more likely to develop diabetes and cardiovascular diseases at an earlier age [5].

Central obesity, in particular, is a critical determinant of insulin resistance and has a multifactorial etiology, involving genetic predisposition and environmental influences during childhood and adolescence. Recent studies have identified adipose tissue as an active endocrine organ capable of secreting various hormones and cytokines, collectively termed adipocytokines. Among these, adiponectin, leptin, tumor necrosis factor-alpha (TNF-α), adipsin, and resistin are of notable significance [6]. Leptin plays a crucial role in regulating energy balance and body weight by modulating appetite and glucose metabolism. Secreted primarily by adipocytes, leptin levels are directly proportional to total body fat. Its primary target is the mediobasal nucleus of the hypothalamus, where it suppresses appetite. Under normal physiological conditions, leptin correlates positively with body fat mass, serving as a satiety signal to reduce food intake and increase energy expenditure. However, in obesity, a paradoxical state of leptin resistance often develops, characterized by elevated leptin levels yet impaired biological response, contributing to metabolic dysfunction and insulin resistance. Leptin deficiency or resistance can result in excessive food intake, obesity, and diabetes [7].

The relationship between leptin and T2DM remains complex and varies across populations and study designs. While several studies have reported elevated leptin levels in individuals with T2DM compared to non-diabetic controls [8,9], these findings predominantly pertain to adult-onset diabetes and populations with varying disease durations. Emerging evidence suggests that leptin dynamics may differ in youth-onset T2DM. In adolescents with T2DM, lower leptin levels were seen compared to obese non-diabetic controls, raising questions about whether relative hypoleptinemia contributes to the pathogenesis of youth-onset T2DM [10]. In previous studies involving Bangladeshi participants, no significant difference in leptin in young participants with or without dysglycemia or gestational diabetes was observed [11,12]. Given the distinct pathophysiological features of youth-onset T2DM compared to adult-onset T2DM, the relationship between leptin levels and disease progression may also differ. Additionally, leptin levels may vary with the duration and severity of diabetes, yet very few studies have examined leptin concentrations in newly diagnosed cases.

Given these inconsistencies and knowledge gaps, further research is warranted to elucidate the role of leptin in youth-onset T2DM. The present study aims to measure and compare circulating leptin levels between individuals with newly diagnosed youth-onset phenotypical T2DM and those with normal glucose tolerance (NGT). Additionally, it explores the associations of leptin with markers of obesity, glycemic parameters, and insulin resistance to better understand its metabolic implications.

## Materials and methods

Study design and subjects

This cross-sectional study involved 36 youth with newly diagnosed diabetes mellitus (DM) (clinically diagnosed T2DM, age range of 13-30 years, both male and female) and an equal number of age-matched young individuals with NGT screened by 75-g two-sample oral glucose tolerance test (OGTT) following American Diabetes Association (ADA) diagnostic criteria for DM [13], attending or referred to the young diabetes clinic, Department of Endocrinology, Bangladesh Medical University (BMU), Dhaka, Bangladesh, during June 2017 to December 2018. Here, we defined "youth" based on a commonly accepted range for youth-onset T2DM, as T2DM is rare before the onset of puberty and considered adult-onset if diagnosed after 30 years. Clinical diagnosis of T2DM was based on the presence of suggestive features like obesity, acanthosis nigricans, family history, and absence of ketosis at presentation. Subjects were excluded if they were pregnant, presented with acute critical illness or any infection within the past month, or had pre-existing chronic medical conditions (including a history or documented evidence of cardiac, hepatic, renal disease, chronic infections, or malignancy). In addition, those who had drug-induced diabetes or diabetes secondary to other diseases were also excluded. The control group consists of young individuals who voluntarily attend the young diabetes clinic to measure their glycemic status and were found to have NGT according to the OGTT.

The sample size was calculated by the formula:

$$n = (Z_\alpha + Z_\beta)^2 \times \frac{(\sigma_1^2 + \sigma_2^2)}{(\mu_1 - \mu_2)^2}$$

Where μ_1 _= mean of leptin in diabetes, σ_1 _= SD of leptin in diabetes, μ_2 _= mean leptin in NGT, σ_2 _= SD of leptin in NGT, Z_α_ = 1.96 at 95% confidence level, and Z_β _= 1.28 at 90% power. Using the leptin levels in a previous study [14], the calculated sample size was 46 for each group. However, the study could include 36 participants in either arm.

Study procedure

Demographic and clinical data were recorded, and anthropometric measurements were done on the same day of the OGTT. Fasting venous blood was collected for measurement of leptin and insulin levels. Body mass index (BMI) was calculated and dichotomized to non-obese (including normal weight and underweight; for adults, BMI <23 kg/m^2^, for children <85^th^ percentile) and obese (including overweight and obesity; for adults, BMI ≥23 kg/m^2^, for children ≥85^th^ percentile). Central obesity was defined as a waist circumference (WC) >90 cm for males and >80 cm for females in adults [15]. For children, a WC of >75th percentile was labeled as centrally obese [16]. Those aged ≤18 years were regarded as children. Homeostasis model assessment-insulin resistance (HOMA-IR) was calculated as follows: HOMA-IR = (fasting plasma insulin × fasting plasma glucose)/22.5 [17].

Analytical methods

Plasma glucose was estimated by the glucose-oxidase method using the Dimension EXL 200 Integrated Chemistry System (Siemens, Munich, Germany), and glycosylated hemoglobin (HbA1c) was measured using the National Glycohemoglobin Standardization Program (NGSP)-certified Bio-Rad D-10^TM^ Hemoglobin A1c Program 220-0101 (Bio-Rad, Hercules, CA). The circulating levels of leptin were measured by the DRG Leptin ELISA Kit (DRG International, Inc., Springfield, NJ). Serum insulin levels were measured by chemiluminescent immunoassay method using Access Immunoassay System (REF-33410) (Beckman Coulter, Inc., Brea, CA). A quality control (QC) sample and a fixed standard were used in every assay run.

Ethical aspects

Informed written consent or assent was obtained from each participant and their guardians (where needed). The protocol was approved by the Institutional Review Board of Bangabandhu Sheikh Mujib Medical University (No.: BSMMU/2018/13312).

Statistical analysis

All data were processed using IBM SPSS Statistics for Windows, version 22.0 (IBM Corp., Armonk, NY). Results were described in frequencies with percentages for qualitative values and mean (±SD/SE) or median (with IQR) for quantitative values with normal or skewed distribution, respectively. Variables were compared between DM and NGT groups using the independent sample t-test, Mann-Whitney U test, or chi-squared test. DM and NGT groups were subdivided into obese and non-obese groups to compare leptin using the Kruskal-Wallis test with post-hoc pairwise comparison. Spearman’s correlation analysis was used to analyze the correlation of leptin with clinical and biochemical variables. Both univariate and multivariate logistic regression analyses were performed to see the predictive association of age, gender, BMI, WC, glycemic status, and HOMA-IR with the elevated leptin level (>75th percentile).

## Results

The characteristics of the study participants and comparison between the DM and NGT groups are shown in Table 1.

**Table 1:** Demographic and clinical characteristics of the subjects (n = 72). Within parentheses are percentages over the column total if not mentioned otherwise. Significance values stand for comparison between DM and NGT groups by independent sample t-test, Mann-Whitney U test, or chi-squared test, as applicable. * Family history of DM in first-degree relatives. † Non-obese: BMI <23 kg/m^2^ or <85th percentile, obese BMI ≥23 kg/m^2^ or ≥85th percentile. ‡ Obese: WC >90 cm for males, >80 cm for females in adults and >75th percentile in children. DM: diabetes mellitus; NGT: normal glucose tolerance; BMI: body mass index; WC: waist circumference; SBP: systolic blood pressure; DBP: diastolic blood pressure; FPG: fasting plasma glucose; 2-hr PG: two-hour plasma glucose; IQR: interquartile range; HbA1c: glycosylated hemoglobin; HOMA-IR: insulin resistance by homeostasis model assessment.

Variables	DM (n = 36)	NGT (n = 36)	Test statistic	p
Age (years; mean ± SD)	24.9 ± 4.4	23.2 ± 4.7	t = -1.521	0.133
Gender				
Female	17 (47.2)	27 (75.0)	ꭓ = 5.844	0.016
Male	19 (52.8)	9 (25.0)		
*Family history of DM	20 (55.6)	18 (50.0)	ꭓ = 0.223	0.637
BMI (kg/m^2^; mean ± SD)	24.1 ± 6.1	24.7 ± 5.1	t = 0.437	0.699
†BMI categories				
Non-obese	18 (50.0)	12 (33.3)	ꭓ = 2.057	0.151
Obese	18 (50.0)	24 (66.7)		
‡WC categories				
Non-obese	20 (55.6)	21 (58.3)	ꭓ = 0.057	0.812
Obese	16 (44.4)	15 (41.3)		
SBP (mm Hg; mean ± SD)	115.0 ± 14.5	112.0 ± 7.6	t = 1.019	0.313
DBP (mm Hg; mean ± SD)	76.9 ± 8.7	71.3 ± 7.4	t = 2.986	0.004
FPG (mmol/L; mean ± SD)	13.4 ± 6.0	4.9 ± 0.5	t = 8.360	<0.001
2-hr PG (mmol/L; mean ± SD)	19.7 ± 7.6	6.3 ± 1.3	t = 10.529	<0.001
HbA1c (%; mean ± SD)	9.6 ± 2.7	5.5 ± 0.4	t = 8.819	<0.001
Fasting insulin (μIU/ml; median and IQR)	10.0 (5.9-18.2)	5.4 (4.2-8.1)	u = 930	0.001
HOMA-IR (median and IQR)	6.1 (3.5-11.3)	1.1 (0.9-1.7)	u = 1194	<0.001

There was no significant difference in respect of age, family history of DM, BMI, WC, and systolic blood pressure (SBP) between the DM and NGT groups. There was a female predominance in the NGT group (p = 0.016), whereas diastolic blood pressure (DBP) was higher in the DM group (p < 0.05 for all).

**Figure 1:**
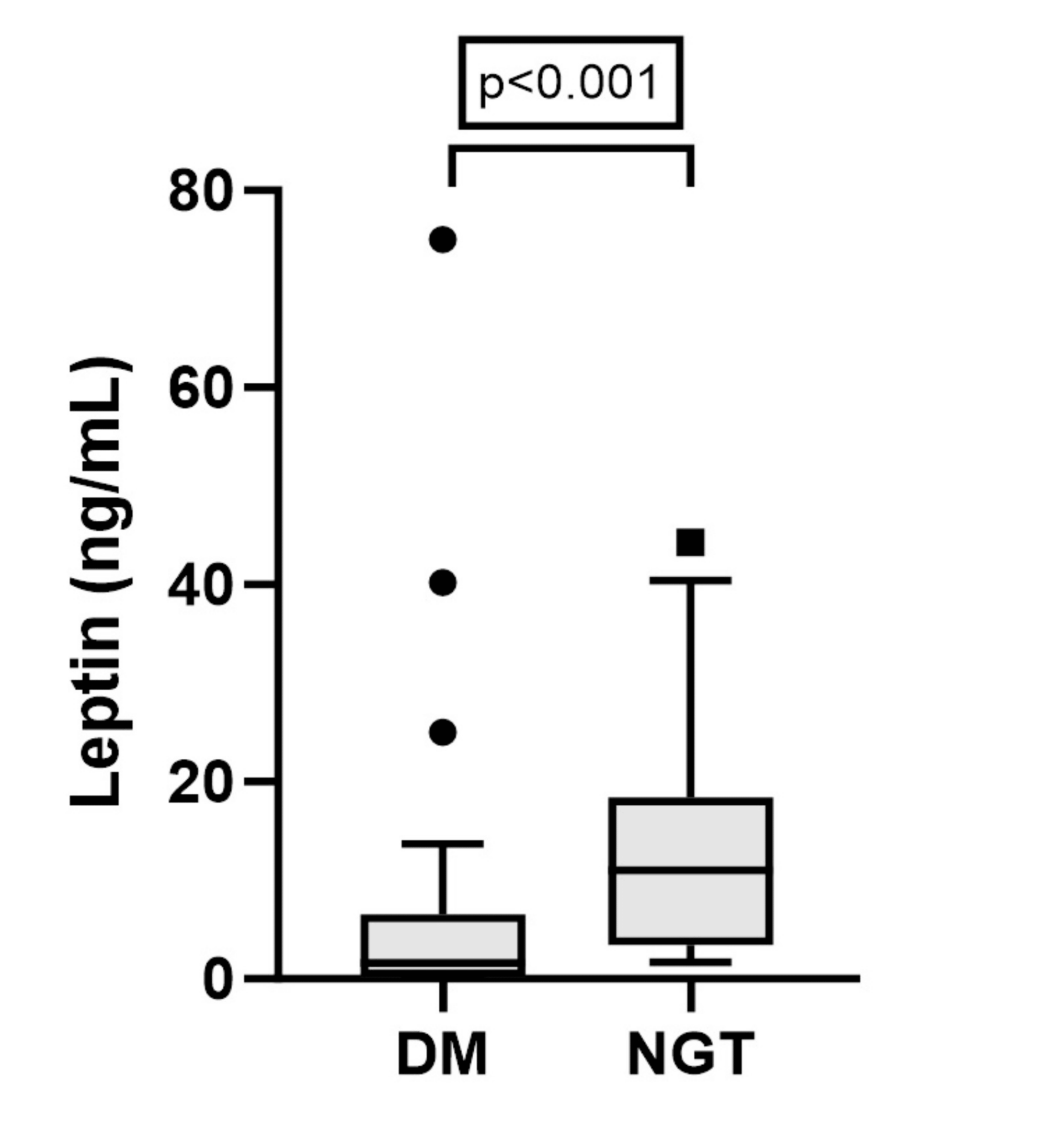
Comparison of leptin between DM (n = 36) and NGT (n = 36) individuals. P-value by Mann-Whitney U test. DM: diabetes mellitus; NGT: normal glucose tolerance.

Leptin was significantly lower in the DM group than in the NGT group (DM vs. NGT: leptin (median & IQR) = 1.6 (0.2-6.6) vs. 11.0 (3.5-18.4), p < 0.001) (Figure 1).

**Figure 2:**
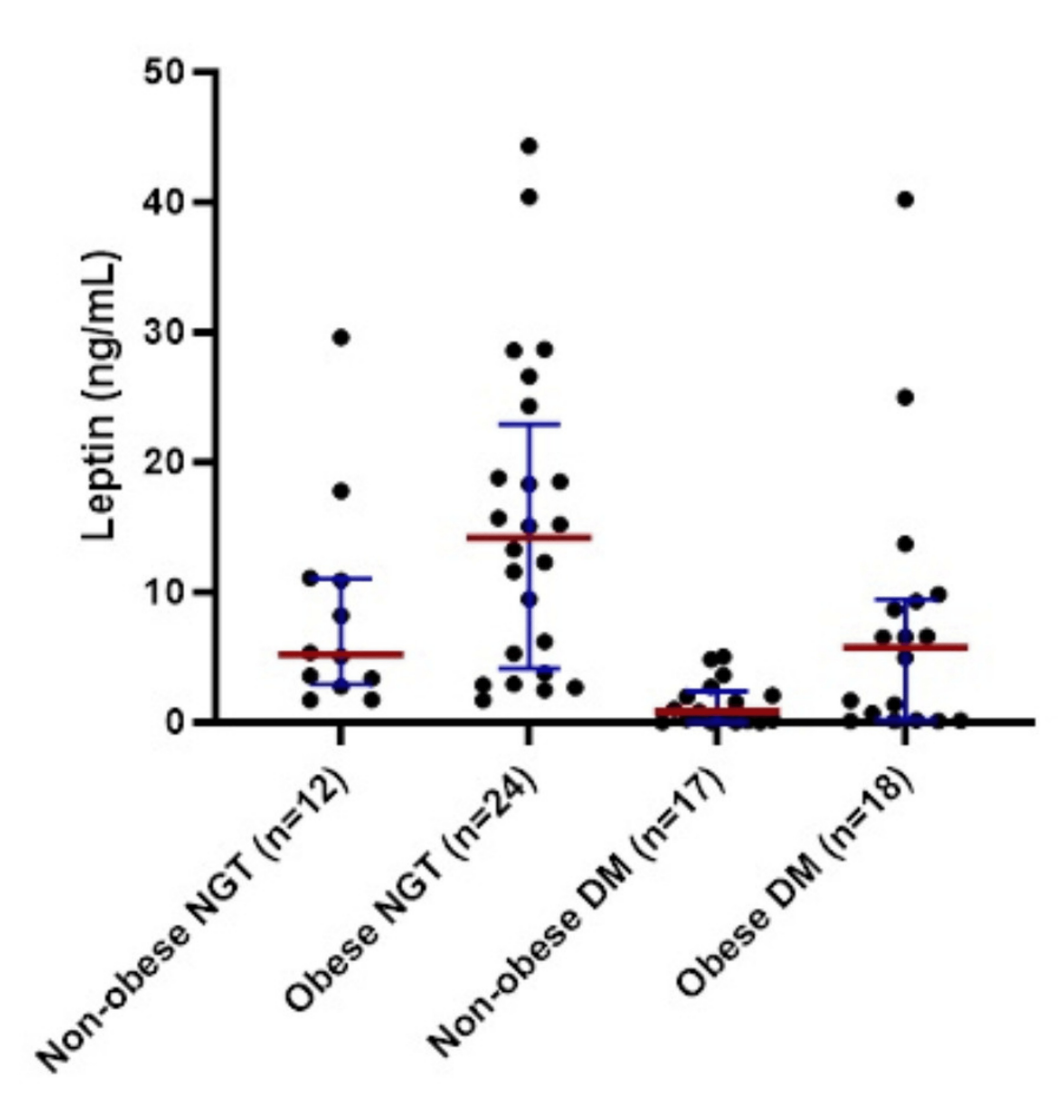
Leptin levels in different BMI categories in participants with DM and NGT. Lines correspond to the median and interquartile range for the leptin level of the subgroup. * One participant (non-obese DM) with an extreme leptin value was excluded from the analysis. Between-groups mean rank difference, p < 0.001 by Kruskal-Wallis test. Post-hoc pairwise comparison revealed p < 0.05 for comparison between non-obese DM vs. non-obese NGT/obese NGT and obese DM vs. obese NGT. Non-obese: BMI <23 kg/m2 or <85th percentile, obese BMI ≥23 kg/m2 or ≥85th percentile. NGT: normal glucose tolerance; DM: diabetes mellitus.

The participants were categorized as non-obese NGT, obese NGT, non-obese DM, and obese DM for comparisons of leptin levels. The between-groups mean rank of leptin levels was significantly different (p < 0.001). Post-hoc pairwise comparison revealed a lower mean rank of leptin in non-obese DM individuals in comparison to both non-obese and obese NGT individuals, while the mean rank of leptin in obese DM individuals was lower than that of obese NGT individuals (Figure 2).

Table 2 presents the correlations between various clinical and biochemical variables and leptin levels among the study participants stratified by glycemic status.

**Table 2:** Correlation of serum leptin levels with different variables. P-value obtained by Spearman's correlation test. DM: diabetes mellitus; NGT: normal glucose tolerance; BMI: body mass index; WC: waist circumference; FPG: fasting plasma glucose; 2hPG: two-hour plasma glucose; HbA1c: hemoglobin A1c; HOMA-IR: insulin resistance by homeostasis model assessment; FI: fasting insulin.

Variables	All participants (n = 72)		DM (n = 36)		NGT (n = 36)	
	ρ	p	ρ	p	ρ	p
Age	-0.20	0.098	0.04	0.839	-0.22	0.198
BMI	0.45	<0.001	0.47	0.004	0.40	0.017
WC	0.28	0.019	0.48	0.003	0.34	0.041
FPG	-0.49	<0.001	-0.13	0.455	0.04	0.828
2hPG	-0.37	0.001	-0.08	0.648	0.29	0.084
HbA1c	-0.39	0.001	-0.06	0.742	0.09	0.598
HOMA-IR	-0.25	0.032	0.24	0.164	0.18	0.296
FI	-0.00	0.989	0.27	0.114	0.18	0.298

There was a significant positive correlation of both BMI and WC with leptin levels in all participants (BMI: ρ = 0.45, p < 0.001; WC: ρ = 0.28, p = 0.019), as well as within the DM (BMI: ρ = 0.47, p = 0.004; WC: ρ = 0.48, p = 0.003) and NGT groups (BMI: ρ = 0.40, p = 0.017; WC: ρ = 0.34, p = 0.041). All the glycemic measurements, including fasting plasma glucose (FPG), plasma glucose two hours after glucose load (2hPG), and HbA1c, as well as HOMA-IR, exhibited a significant negative correlation with the leptin levels when analyzed within all participants, but not separately in either DM or NGT groups. No significant correlation of leptin with age or fasting insulin level was found in any of the groups.

In the logistic regression analysis for predictors of elevated leptin levels (above the 75th percentile, cutoff: 13.60 ng/mL) among the study participants, significant associations were observed with gender, BMI, WC, and glycemic status in the univariate model. However, in the multivariate model (adjusted for age and gender), BMI and glycemic status remained independent predictors of elevated leptin (Table 3). A higher BMI was associated with increased odds of elevated leptin levels, whereas DM was associated with decreased odds of elevated leptin.

**Table 3:** Logistic regression analysis for the prediction of elevated leptin level (above the 75th percentile, cutoff: 13.60 ng/mL) among the participants (n = 72). OR: odds ratio; CI: confidence interval; BMI: body mass index; DM: diabetes mellitus; NGT: normal glucose tolerance; HOMA-IR: insulin resistance by homeostasis model assessment.

Variable	Univariate		Multivariate	
	OR (95% CI)	p-value	OR (95% CI)	p-value
Age (each year increase)	0.9 (0.8-1.0)	0.058	0.9 (0.8-1.1)	0.204
Female gender (in comparison to male)	4.3 (1.1-16.6)	0.034	4.0 (0.7-23.5)	0.121
BMI (each kg/m^2^ increase)	1.3 (1.1-1.5)	0.001	1.3 (1.1-1.6)	0.001
Waist circumference (each cm increase)	1.1 (1.0-1.2)	0.005	-	-
DM (in comparison to NGT)	0.2 (0.1-0.7)	0.010	0.1 (0.0-0.8)	0.030
HOMA-IR (each unit increase)	0.9 (0.8-1.1)	0.281	-	-

## Discussion

Leptin is a key adipokine involved in the regulation of energy intake and expenditure, playing a crucial role in obesity and insulin resistance. However, its role in youth-onset T2DM remains unclear. The current study aimed to measure and compare leptin levels between individuals with phenotypical youth-onset T2DM and those with NGT. An equal number of individuals with DM and age-matched controls were enrolled in this study. Both groups were statistically comparable in terms of positive family history of DM among first-degree relatives and the distribution across BMI and WC categories. The findings revealed that leptin levels were significantly lower in the T2DM group compared to the NGT group. Notably, non-obese DM individuals had lower leptin levels than both non-obese and obese NGT individuals, while obese DM individuals had lower leptin levels than their obese NGT counterparts. There was a positive correlation of leptin with obesity markers (BMI and WC) and a negative correlation with glycemic parameters (FPG, 2hPG, and HbA1c) as well as insulin resistance (HOMA-IR). BMI and glycemic status were independent predictors of elevated leptin levels.

Leptin has been extensively studied in recent years as a potential marker of insulin resistance, a key component of diabetes and other metabolic disorders, including metabolic syndrome and polycystic ovary syndrome (PCOS) [18]. Given that leptin levels are typically elevated in overweight and obese individuals, and that obesity is a major risk factor for insulin resistance and T2DM, it was expected that participants with youth-onset T2DM would exhibit higher leptin levels compared to those with NGT. Most of the studies on leptin levels in T2DM compared to healthy controls found higher leptin levels in T2DM patients [8,9]. However, the findings of the present study differed from these observations. Many previous studies that reported higher leptin levels in T2DM included participants of varying ages and did not specifically recruit newly diagnosed individuals. It is observed that the leptin level rises with longer duration of diabetes [19]. Additionally, BMI matching between diabetic and control groups was often not considered. In contrast, a recent study found that adolescents with T2DM had significantly lower leptin levels compared to non-diabetic controls, leading the authors to suggest that relative hypoleptinemia in obese adolescents with T2DM may contribute to disease development [10]. Similarly, a study conducted in an indigenous African population reported lower circulating leptin levels in diabetic individuals compared to age- and BMI-matched controls [20]. The authors hypothesized that this difference might be attributed to variations in fat distribution; however, fat distribution was not assessed in that study.

Although leptin levels exhibited a positive correlation with BMI and WC, they were negatively correlated with glycemic parameters, suggesting that, at least in newly diagnosed cases, the development of DM may have a suppressive effect on leptin levels. The underlying mechanism remains unclear. One possible explanation is that weight loss often occurs at the time of DM diagnosis. In this study, nearly half of the participants with DM had HbA1c levels ≥10%, indicating poor glycemic control, which may have led to a reduction in fat mass and, consequently, lower leptin levels. However, BMI and WC were comparable between participants with DM and those with NGT, suggesting that additional factors may contribute to the observed leptin reduction in youth-onset phenotypical T2DM. Given the distinct characteristics of youth-onset T2DM, including rapid β-cell dysfunction and an early onset of complications, the precise pathophysiological mechanisms underlying this condition remain incompletely understood. Furthermore, while leptin levels correlated with obesity markers (BMI and WC), no significant correlation was observed with insulin resistance, as measured by HOMA-IR. It is also unclear whether there are some limitations of HOMA-IR in youth, especially in the early stage of the disease. Nevertheless, this finding suggests that leptin may not have a direct causal role in insulin resistance but rather that both leptin and insulin resistance may result from common pathways linked to increasing adiposity. A previous study found a positive correlation of leptin with insulin resistance in participants with abnormal glucose tolerance (AGT) [11]. However, only a minority of the participants with AGT had DM, and the group was predominantly comprised of individuals with prediabetes.

The strengths of this study include the enrollment of age-matched controls who had comparable BMI and WC with youth-onset T2DM participants. As the study involved only newly diagnosed T2DM, the confounding effects of disease duration on leptin levels could be overcome. The study’s focus on a distinct population with youth-onset phenotypical T2DM provides insights into the metabolic characteristics of this group, highlighting potential differences from adult-onset T2DM. The limitations of this study include the reliance on clinical characteristics for diabetes classification, making it difficult to exclude slow-progressing immune-mediated diabetes or monogenic forms such as maturity-onset diabetes in young (MODY). Additionally, fat distribution, a potential factor influencing leptin levels, was not assessed. The cross-sectional design limits the ability to establish causal relationships between leptin levels, obesity, and glycemic status. The study was unable to include the desired number of samples, which reduces its statistical power. The lack of pubertal assessment in the case of adolescent participants and female predominance in the control group may further affect the analytic inference.

## Conclusions

In conclusion, leptin levels were significantly lower in individuals with newly diagnosed youth-onset T2DM compared to those with NGT, despite a positive correlation with obesity markers. Additionally, a negative correlation was observed between leptin levels and glycemic parameters, suggesting an association between reduced leptin levels and impaired glucose metabolic function. While these findings suggest the potential involvement of leptin in youth-onset T2DM, further research is needed to elucidate the dynamics of leptin metabolism in this population. Moreover, the relationship between leptin and insulin resistance warrants further exploration to clarify their roles in the pathophysiology of youth-onset diabetes.
